# Emerging therapies for severe asthma

**DOI:** 10.1186/1741-7015-9-102

**Published:** 2011-09-06

**Authors:** Neil C Thomson, Rekha Chaudhuri, Mark Spears

**Affiliations:** 1 Respiratory Medicine, Institute of Infection, Immunity, & Inflammation, University of Glasgow, Glasgow, G12 OYN UK

## Abstract

Many patients with asthma have poorly controlled symptoms, and particularly for those with severe disease, there is a clear need for improved treatments. Two recent therapies licensed for use in asthma are omalizumab, a humanized monoclonal antibody that binds circulating IgE antibody, and bronchial thermoplasty, which involves the delivery of radio frequency energy to the airways to reduce airway smooth muscle mass. In addition, there are new therapies under development for asthma that have good potential to reach the clinic in the next five years. These include biological agents targeting pro-inflammatory cytokines such as interleukin-5 and interleukin-13, inhaled ultra long-acting β_2_-agonists and once daily inhaled corticosteroids. In addition, drugs that block components of the arachidonic acid pathway that targets neutrophilic asthma and CRTH2 receptor antagonists that inhibit the proinflammatory actions of prostaglandin D_2 _may become available. We review the recent progress made in developing viable therapies for severe asthma and briefly discuss the idea that development of novel therapies for asthma is likely to increasingly involve the assessment of genotypic and/or phenotypic factors.

## Introduction

**Figure 1:**
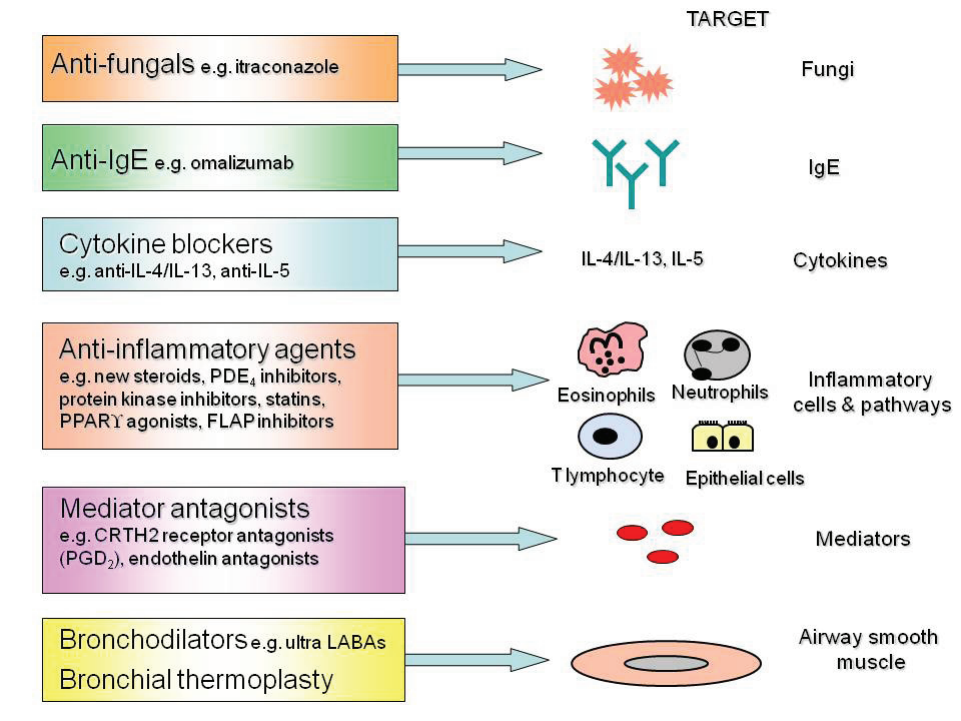
**Potential targets for selected novel therapies for treatment resistant asthma**. The figure summarizes targets for a selection of therapies that are recently licensed or under clinical development for patients with severe treatment resistant asthma. Abbreviations: CRTH2, chemoattractant receptor-homologous molecule expressed on Th2 cells; FLAP, lipoxygenase-activating protein; IL-, interleukin-; PPAR, proliferator-activated receptor; PDE, phosphodiesterase; PGD_2_, prostaglandin D_2_.

Asthma is a chronic inflammatory disease of the airways that affects over 300 million individuals worldwide [1]. The majority of adults with asthma have mild or moderate disease that can be controlled by inhaled corticosteroids either alone or in combination with inhaled long-acting ß_2 _agonist bronchodilators [1-3]. Questionnaire surveys however indicate that a considerable proportion of these patients [4], as well as most with severe asthma [5], or who are cigarette smokers [6,7] have poorly controlled asthma. Systematic evaluation can help identify patients with severe asthma from those with difficult-to-treat asthma due to poor adherence, untreated co-morbidities, dysfunctional breathing or psychological problems [8,9]. For patients with severe asthma, which accounts for 5% to 10% of cases [10], there is a need for improved therapies [10-12]. This mini-review focuses on biological agents, new inhaled long-acting bronchodilators and corticosteroids, arachidonic acid pathway blockers, bronchial thermoplasty plus a range of other anti-inflammatory agents that have been recently licensed or are at an advanced stage of development for patients with severe asthma (Figure 1). In addition, we briefly discuss the idea that the development of novel therapies for asthma is likely increasingly to involve the assessment of genotypic and/or phenotypic factors.

### Biological agents

The first and as yet only biological agent licensed for the treatment of asthma is omalizumab, a humanized monoclonal antibody that binds circulating IgE antibody, preventing it from binding to its specific high-affinity receptor on mast cells and basophils [13]. In patients with allergic asthma, omalizumab treatment improves symptoms and reduces exacerbations [14,15]. Clinical trials are also underway to assess the efficacy of omalizumab in non-allergic asthma and in combination with specific allergen immunotherapy, with the aim of reducing systemic allergic reactions [16]. The adverse effect profile of omalizumab is generally good [17] although preliminary data from a five-year safety study has raised concerns about a trend for increased cardiovascular events and further confirmation is awaited [18,19].

A number of biological agents have been developed to target cytokines thought to play an important role in asthma pathogenesis [20,21], including monoclonal antibody blockers of TNF-α, IL-5, IL-4 and IL-13. Unfortunately despite some promise shown in early small clinical studies with the soluble TNF-α receptor blocker, etanercept, in severe asthma [22,23], larger studies with golimumab [24] and etanercept [25] have not confirmed a consistent effect. Overall, when combined with concerns over increased risk of severe infections and malignancies with treatment [24] it is unlikely that TNF-α receptor blockers will be developed further for the treatment of asthma.

**Figure 2:**
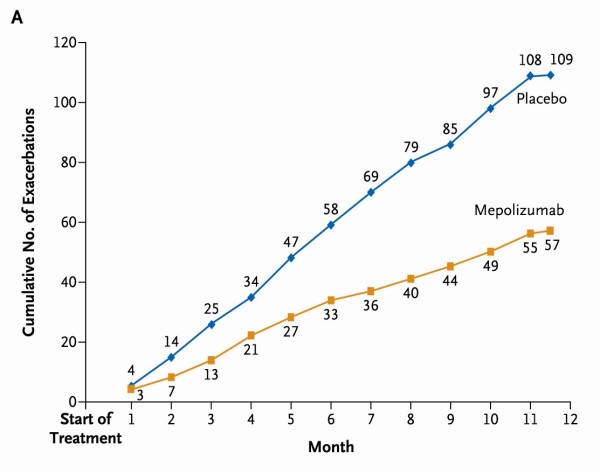
**Cumulative number of severe exacerbations in each study group over the course of 50 weeks treatment with mepolizumab or placebo**. Mean number of exacerbations per subject over the course of the 50-week treatment period was 2.0 in the mepolizumab group, compared with 3.4 in the placebo group (relative risk, 0.57; 95% confidence interval, 0.32 to 0.92; *P *= 0.02). Reproduced from Haldar et al with permission [27]. Copyright (c) Massachusetts Medical Society.

Two recent exploratory studies have examined anti-IL5 monoclonal antibody (mepolizumab) treatment in patients with severe asthma [26,27]. In 61 patients with refractory eosinophilic asthma and a history of recurrent severe exacerbations mepolizumab treatment reduced severe exacerbations [27] (Figure 2) and in 20 patients with severe oral corticosteroid dependent asthma an oral corticosteroid sparing effect was observed [26]. Phase 3 trials are now underway. The relevance of this approach to clinical practice has been debated [28] as possibly only a small proportion of patients with persistent sputum eosinophilia are also concordant with inhaled or oral corticosteroid treatment [29].

A number of clinical trials employing monoclonal antibodies targeting IL-4 and/or IL-13 in asthma are underway [30,31]. Both cytokines exert their actions through the IL-4Rα/IL-13Rα1 receptor complex. Blocking IL-13 binding to the IL-4 receptor α with IMA-638 reduces allergen-induced bronchoconstriction [32]. Pitrakinra, a recombinant protein that binds to IL-4Rα, reduces allergen-induced late responses with few adverse events [33] and is undergoing development as an inhaled medication. A clearer picture of the role of IL-4 and IL-13 blockers in the treatment of severe asthma is likely to emerge over the next few years.

Daclizumab is a humanized monoclonal antibody that binds specifically to the CD25 subunit of the high-affinity IL-2R, and inhibits IL-2 binding and T-cell activation. A pilot study of daclizumab in patients with moderate to severe asthma reported minor improvements in lung function and asthma control [34].

Patients with asthma may be more susceptible to respiratory viruses due to impaired Th_1 _immunity [35] and immunological augmentation with inhaled interferon β to aid anti-viral responses at the time of the exacerbation is currently under assessment.

### New inhaled long-acting bronchodilators and corticosteroids

In addition to the development of novel therapies, refinements in the pharmacological properties of drugs currently used to treat asthma, such as long-acting β_2_-agonists and inhaled corticosteroids is a major focus of the pharmaceutical industry. Inhaled ultra long-acting β_2_-agonists (ultra-LABAs) such as indacaterol, currently licensed for COPD, have a longer half-life than current LABAs and are suitable for once daily administration [36,37]. Fixed combinations of ultra-LABAs with once daily inhaled corticosteroids as well as once daily inhaled corticosteroids alone are at an advanced stage of development for the treatment of asthma. Once daily administration should be more convenient for patients and may improve adherence. New inhaled long-acting antimuscarinic agents (LAMAs) agents such as aclidinium, may also have a role in the treatment of severe asthma associated with persistent airflow obstruction [38,39]. Non-steroidal selective glucocorticoid receptor modulators are in development with the aim of improving the therapeutic ratio of corticosteroids by dissociating transactivation, which is associated with the adverse effects of corticosteroids, from the beneficial effect of transrepression [40-43].

### Arachidonic acid pathway blockers

Pro-inflammatory cysteinyl leukotrienes (LTs) are synthesized from arachidonic acid by 5-lipoxygenase (LO) and 5-lipoxygenase-activating protein (FLAP) in inflammatory airway cells. In addition to inhibiting the production of the cysteinyl LTs, 5-LO and FLAP inhibitors such as GSK-2190915 [44] prevent the formation of LTB_4_, which may be of value in neutrophilic asthma. Prostaglandin (PG)D_2 _is released from mast cells and other inflammatory cells and elevated bronchoalveolar lavage concentrations of PGD_2 _are reported in severe asthma [45]. PGD_2 _activates the CRTH2 receptor (chemoattractant receptor-homologous molecule expressed on Th_2 _cells) resulting in inflammation and a number of antagonists of the CRTH2 receptor are being assessed for the treatment of asthma [46].

### Bronchial thermoplasty

**Figure 3:**
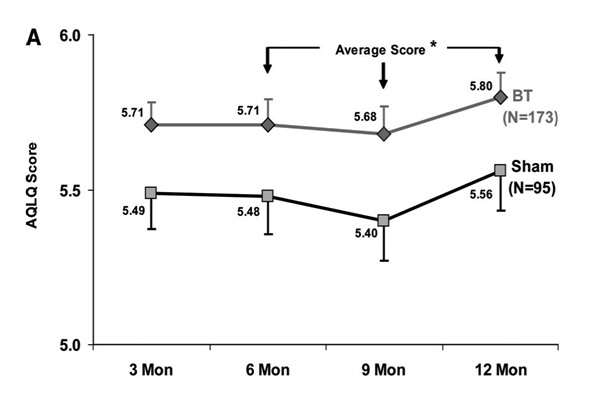
**Total Asthma Quality of Life Questionnaire (AQLQ) score over 12 months after treatment with bronchial thermoplasty (BT) (diamonds) or sham control (squares) in the per protocol population**. *Posterior probability of superiority = 97.9%. Reprinted with permission of the American Thoracic Society. Copyright ^© ^American Thoracic Society [54] Official Journal of the American Thoracic Society.

Bronchial thermoplasty involves the delivery of radio frequency energy to the airways by flexible bronchoscopy with the aim of reducing airway smooth muscle mass and responsiveness in asthma [47-50]. Initial clinical studies including the Asthma Intervention Research (AIR)1 trial found that bronchial thermoplasty reduces exacerbations and improves morning peak expiratory flow and symptoms in patients with severe asthma [51-53]. The AIR2 trial reported the results of a comparison with sham bronchial thermoplasty in 288 adult subjects with severe asthma [54]. Bronchial thermoplasty resulted in improvements from baseline in Asthma Quality of Life Questionnaire (AQLQ) scores compared with sham (bronchial thermoplasty, 1.35 versus sham, 1.16), with 79% of bronchial thermoplasty and 64% of sham subjects achieving changes in AQLQ of 0.5 or greater (Figure 3). It is of interest that the sham bronchial thermoplasty was associated with a large increase in AQLQ scores. In the post-treatment period, the bronchial thermoplasty group also experienced fewer severe exacerbations and emergency department visits. However bronchial thermoplasty was associated with a short-term increase in asthma-related morbidity. Long-term five-year safety data for patients recruited to the AIR1 trial [52] reported absence of clinical complications and maintenance of stable lung function [55]. In 2010 bronchial thermoplasty was approved by the United States Food & Drug Administration (FDA) for the treatment of severe persistent asthma. Future research needs to identify factors that best predict a therapeutic response to bronchial thermoplasty.

### Other anti-inflammatory drugs

A number of novel drugs under development may prove to have a role in the management of asthma. Phosphodiesterase (PDE)_4 _inhibitors have immunomodulatory effects over a number of inflammatory cells potentially relevant to the treatment of severe asthma [56]. High doses of phosphodiesterase (PDE)_4 _inhibitors may be necessary to treat severe asthma, and gastro-intestinal side effects may limit their use [56-58], although inhaled PDE_4 _inhibitors may improve their therapeutic index [59,60]. Inhibition of protein kinases such as p38 mitogen-activated protein kinase (MAPK) and other tyrosine kinases involved in cellular signalling of pro-inflammatory cytokines may have a role in the treatment of severe asthma [61-63]. For example, a phase 3 study evaluating a tyrosine kinase inhibitor of the c-KIT receptor masitinib commenced recently.

Several drugs licensed for treating other conditions may also have a role in the management of asthma. In a randomized controlled trial of 58 patients with severe asthma with fungal sensitization (SAFS) [64] the oral anti-fungal drug itraconazole administered for 32 weeks resulted in improvement in AQLQ scores [65]. However the risk of adrenal suppression with long-term treatment with itraconazole in patients also receiving inhaled corticosteroids has led to some caution in adopting this management strategy [66,67]. Peroxisome proliferator-activated receptor-γ (PPARγ) agonists exert anti-inflammatory effects on eosinophilic and neutrophilic infiltration of the lungs of experimental animals [68]. A proof of concept study using the PPARγ agonist rosiglitazone, demonstrated bronchodilator effects in mild to moderate smokers with asthma [69] and further clinical trials of oral PPARγ agonists are underway in severe asthma. Statins have pleiotropic anti-inflammatory effects potentially relevant to the treatment of asthma [70-76]. Short-term treatment of patients with mild to moderate asthma with statins does not improve symptoms or lung function [77-80], although AQLQ scores improve in asthmatic smokers [80]. The small peptide endothelin causes bronchoconstriction and may contribute to airway remodelling in asthma [81,82]. A 12-month clinical trial of the endothelin receptor antagonist sitaxsentan is underway in patients with severe asthma.

### Factors influencing the response to novel therapies

Genotypic and phenotypic factors can influence the response to drug treatment for asthma [23,26,27,83]. For example, adrenergic β_2_-receptor polymorphisms, particularly variants at position 16 (Gly16Arg) and 27 (Gln27Glu), have been associated with impaired bronchodilator response to short and long-acting β_2_-receptor agonists and naturally occurring mutations in the promoter of 5-lipoxygenase gene (ALOX5) may influence the clinical response to drugs modifying the 5-lipoxygenase pathway [84-86]. Several inflammatory phenotypes, which have been identified mainly on the basis of induced sputum cell profiles, influence the response to drug treatment [87,88]. For example, sputum eosinophilia predicts corticosteroid responsiveness [89-93] and the response to the anti-IL-5 blocker mepolizumab in severe asthma [26,27]. Non-eosinophilic asthma, a term used to describe an absence of raised numbers of inflammatory cells (also known as paucigranulocytic inflammation) or neutrophilic inflammation, responds less well to inhaled corticosteroids [91-93]. Macrolides may be effective in neutrophilic asthma [94]. Th_2_-high asthma, as defined by gene expression analyses of airway cells, predicts an improvement in lung function with inhaled corticosteroids in patients with mild to moderate disease, whereas patients with Th_2_-low asthma respond poorly to inhaled corticosteroids [95]. The development of novel therapies for severe asthma in the future is likely to involve genotypic and/or phenotypic assessment to identify patients who will gain the most from a specific intervention.

### Conclusions and future directions

Omalizumab, a humanized monoclonal antibody that binds circulating IgE antibody, and bronchial thermoplasty, where radio frequency energy is delivered to the airways to reduce airway smooth muscle mass are valuable additional therapies for the management of severe asthma. There is a need to identify new therapies that are effective and safe and target sub-phenotypes of asthma. Of therapies currently under development, biological agents directed at blocking pro-inflammatory cytokines such as interleukin-5 and interleukin-13, ultra long-acting β_2_-agonists and once daily inhaled corticosteroids as well as drugs blocking components of the arachidonic acid pathway such as FLAP inhibitors and CRTH2 receptor antagonists have the greatest potential to reach the clinic. In the future, both genotypic and phenotypic factors are likely to guide the choice of intervention in each individual with severe asthma.

## List of abbreviations

AIR trial: Asthma Intervention Research trial; AQLQ: asthma quality of life questionnaire; CRTH2: chemoattractant receptor-homologous molecule expressed on Th_2 _cells; FDA: U.S. Food & Drug Administration; FLAP: lipoxygenase-activating protein; IL-: interleukin; LAMAs: long-acting antimuscarinic agents; LO: lipoxygenase; LTs: leukotrienes; MAPK: mitogen-activated protein kinase; PPAR: proliferator-activated receptor; PDE: phosphodiesterase; PGD_2: _prostaglandin D_2; _SAFS: severe asthma with fungal sensitization; TNF: tumor necrosis factor; Ultra-LABAs: ultra long-acting β_2_-agonists.

## Competing interests

NCT received industry-sponsored grant funding from Aerovance, Asthmatx, Glaxo Smithkline, MedImmune Ltd, Novartis, Centocor and Synairgen for participating in clinical trials.

## Authors' contributions

NCT conceived the article and prepared the initial draft of the manuscript. All authors drafted further versions of the manuscript and approved the final version.

## Pre-publication history

The pre-publication history for this paper can be accessed here:

http://www.biomedcentral.com/1741-7015/9/102/prepub
